# The pharmaceutical excipient PEG400 affect the absorption of baicalein in Caco‐2 monolayer model by interacting with UDP‐glucuronosyltransferases and efflux transport proteins

**DOI:** 10.1002/prp2.928

**Published:** 2022-02-11

**Authors:** Siyuan Cao, Min Zhang, Minyan Yuan, Dan Yang, Mei Zhao, Shuo Zhang, Pengjiao Wang, Rongping Zhang, Xiuli Gao

**Affiliations:** ^1^ State Key Laboratory of Functions and Applications of Medicinal Plants and School of Pharmacy Guizhou Medical University Guiyang China; ^2^ Department of Education of Guizhou Center of Microbiology and Biochemical Pharmaceutical Engineering Guiyang China; ^3^ Experimental Animal Center of Guizhou Medical University Guiyang China; ^4^ School of Pharmacy Kunming Medical University Kunming China

**Keywords:** baicalein, baicalein‐6‐O‐β‐d‐glucuronide, baicalein‐7‐O‐β‐d‐glucuronide, Caco‐2 cell, transport proteins, UDP‐glucuronosyltransferases

## Abstract

The bioavailability of drugs is often related to intestinal metabolism and transport mechanisms. In previous studies, pharmaceutical excipients were recognized as inert substances in clinical safety evaluations. However, a large number of studies have shown that pharmaceutical excipients regulate the metabolism and transport of drugs in the body and improve the bioavailability. The pharmaceutical excipient polyethylene glycol 400 (PEG400) as a good solubilizer and surfactant has the potential to improve the bioavailability of drugs. The combined action of UDP‐glucuronosyltransferases (UGTs) and efflux transport proteins is responsible for the intestinal disposition and poor bioavailability of baicalein. Our aim is to study the effect of PEG400 on the absorption of baicalein on the Caco‐2 monolayer, and confirm the interaction of PEG400 with UGTs (UGT1A8 and UGT1A9) and efflux transports. We initially found that baicalein in the Caco‐2 monolayer would be metabolized into glucuronide conjugates BG and B6G under the action of UGT1A8 and UGT1A9 on the endoplasmic reticulum membrane, and then mainly excreted to different sides by acting of MRP and BCRP. The addition of PEG400 significantly accelerated the metabolism of B in Caco‐2 cells and increased the penetration of BG and B6G. Furthermore, PEG400 also significantly decreased the efflux ratio of BG and B6G, which was the evidence of the interaction with the efflux transporters. In the in vitro intestinal microsome regeneration system, low concentration PEG400 decreased the *K*_m_ value of UGT1A8 and UGT1A9 (key enzymes that mediate the production of BG and B6G); high concentration PEG400 enhanced the *V*_max_ value of UGT1A8 and UGT1A9. In conclusion, our results determined that PEG400 interacted with some UGTs and efflux transporters, which were the main factors affecting the absorption of baicalein.

## Graphical abstract

The absorption process of baicalein in a CaCo‐2 monolayer model.

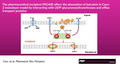

**Abbreviations**
AKP: alkaline phosphatase
B: baicalein
B6G: baicalein‐6‐O‐β‐d‐glucuronide
BCRP: breast cancer resistance protein
BG: baicalein ‐7‐O‐β‐d‐glucuronide
DMSO: dimethyl sulfoxide
ER: efflux ratio
HBSS: hanks’ balanced salt solution
K_m_
: Michaelis constant
MRP: multidrug resistance‐associated proteins
MS: mass spectrometry
MS/MS: tandem mass spectrometry
*P*_app_
: apparent permeability coefficient
P‐GP: P‐glycoprotein
TEER: trans‐epithelial electrical resistance
UGTs: UDP‐glucuronosyltransferases
*V*_max_
: maximum velocity

## 1 INTRODUCTION

The influence of pharmaceutical excipients on pharmacokinetics has been increasingly recognized.^1^, ^2^ The interaction of pharmaceutical excipients with metabolic enzymes and drug transporters is an important part of evaluating the safety and effectiveness of pharmaceutical excipients. Pharmaceutical excipatory polyethylene glycol (PEG400) is a mixture of polycondensation of water and ethylene oxide, commonly used in a variety of pharmaceutical formulations (soft capsules, injections, solid lipid nanoparticles, etc). Studies have shown that PEG400 can promote the activity of phase I drug metabolizing enzyme CYP3A4.^3^ For drug transport protein, PEG400 has be considered as an inhibitor of P‐gp.^4^ The unique biological effects of PEG400 in the in vivo process of drugs require more attention from researchers.

Baicalein (B) is a major flavonoid compound extracted from the traditional Chinese medicine *Scutellaria baicalensis* Georgi. It had been reported to have antibacterial, antitumor, anti‐inflammatory, antiviral, antioxidant pharmacological activities.^5^, ^6^, ^7^, ^8^, ^9^ The small intestine is the main place for drug absorption, and the intestinal mucosa is the first pass for the first pass effect of drugs. In vivo, B will be metabolized into glucuronide conjugates baicalein ‐6‐O‐β‐d‐glucuronide (B6G) and baicalein‐7‐O‐β‐d‐glucuronide (BG) via the phase II metabolic enzyme UDP‐glucuronosyltransferase (UGTs) in the small intestinal mucosa, which restricts baicalein the process of biological absorption. This process is mainly mediated by subtypes UGT1A8 and UGT1A9. In previous studies, we found that PEG400 significantly increased the *C*_max_ and AUC_0‐t_ values of B6G and BG in plasma, and in vitro isoenzyme incubation experiments preliminary confirmed the effect of PEG400 on the enzyme activities of UGT1A8 and UGT1A9.^10^ This suggested that PEG400 interacted with UGT1A8 and UGT1A9.

To a certain extent, in vitro cell experiments can more accurately reflect the effect of PEG400 on drug absorption than integral animal experiments. Caco‐2 cells are derived from human colon adenocarcinoma cell line and have been identified as a perfect in vitro experimental model for the study of drug absorption mechanism.^11^ Studies have shown that cultured on a porous permeable polycarbonate membrane can fuse, differentiate, and form a continuous dense monolayer. Its morphology and function are similar to human small intestinal epithelial cells, and its morphology, marker enzyme expression, uptake, transport, and permeability characteristics are all similar to the small intestinal epithelial cells, it can well simulate the absorption process of the small intestinal epithelial cells.^12^, ^13^ P‐glycoprotein (P‐GP), Multidrug resistance‐associated proteins (MRP), and Breast cancer resistance protein (BCRP) have been identified as highly expressed on Caco‐2 and are the main transport proteins that affect drug efflux.^14^

In this study, we established a Caco‐2 cell model and investigated the effect of PEG 400 on the absorption process of baicalein by UPLC‐MS/MS. In order to further determine the interaction of PEG400 with metabolic enzymes and efflux transporters, specific chemical inhibitor was used to determine the interaction between the PEG400 and transport protein. The intestinal microsome regeneration system was used to determine the interaction between the PEG400 with UGT1A8 and UGT1A9 in a complex enzymatic environment.

## 2 MATERIALS AND METHODS

### 2.1 Chemicals and materials

Polyethylene glycol 400 was obtained from Xiqiao Chemical Co., Ltd. Baicalein‐7‐O‐β‐d‐glucuronide(BG), baicalein and genistein (IS) standards (purity ≥98%) were obtained from China National Institute for the Control of Pharmaceutical and Biological Products. Baicalein‐6‐O‐β‐d‐glucuronide(B6G) standard (purity ≥95%) was obtained from Dalian Medical University. Hank's buffered salt solution (HBSS), phosphate‐buffered saline (PBS), Tris‐HCl buffer solution (PH = 7.4) and Tris‐Tricine‐SDS‐PAGE gel kit were obtained from Solarbio (Beijing Solarbio Science & Technology Co., Ltd.). 100 U/ml of penicillin, 100 μg/ml of streptomycin, fetal bovine serum (FBS), and 0.25% trypsin with ethylenediaminetetraacetic acid (trypsin–EDTA) were obtained from Wiseen. Verapamil, MK571 and KO143 were obtained from MedChemExpress. The human intestine microsomes were obtained from Xenotech. The uridine diphosphate glucuronic acid (UDPGA) (purity ≥95%) was obtained from Quanyang Biotechnology Co., Ltd. The alamethicin (purity ≥98.0%) was obtained from Shanghai Aladdin Biochemical Technology Co., Ltd.

### 2.2 Drug pretreatment

B, BG, and B6G are first dissolved in DMSO. For cell viability assay, samples were diluted with DMEM culture medium (containing 10% (v/v) FBS, 1% (v/v) 100 U/ml penicillin and 100 μg/ml streptomycin) to prepare the different concentrations. For the permeation experiment, samples were diluted with HBSS to prepare the final concentration. For enzyme activity assay, dilute with Tris‐HCl buffered salt solution to prepare different concentrations. The final concentration of DMSO in different samples were controlled below 0.1% (v/v) to ensure the safety to the cells.

### 2.3 Caco‐2 cell culture

Cell culture was performed as previously described.^15^ For transport experiments, Caco‐2 cells from passage number 20–30 were seeded at a density of 2 × 10^5^ cells per well on transwell plate (a solid insert for cell culture contains porous permeable polycarbonate membrane) with polycarbonate membranes using a blood‐counting chamber (QIU JING^®^). A 0.5 ml of culture medium with cells was added to the apical side (AP) and 1.5 ml of blank culture medium was added to the basolateral side (BL). During the incubation period, the medium on the AP and BL sides were changed every 2 days in the first week, and every day after a week until the 21st day.

### 2.4 UPLC‐MS/MS quantitative of samples

Samples were quantified by UPLC‐MS/MS with the liquid chromatography, Thermo UltiMate 3000 Ultra Performance Liquid Chromatography (Thermo Finnigan), and the mass spectrometer used Triple‐quadrupole tandem mass spectrometric detection (Thermo Finnigan) with an ESI source in positive ion mode. Chromatographic separation was performed with Thermo Dim. Hypersil Gold column, 100 × 2.1 mm, 1.9 μm (Thermo Fisher Scientific). Mobile phase A consisted of acetonitrile, while mobile phase B consisted of distillated water containing 0.1% formic acid. The following elution program: the initial of B was 90%, downing from 90% B to 27% B in 0–9 min, backing to 90% B in 9.1 min and maintaining 2.9 min. The total flow rate of the mobile phases was 0.3 ml/min. The auto‐sampler was conditioned at 10°C and the injection volume was 10 µl. Quantification was performed using single reaction monitoring (SRM) of the transitions of *m*/*z* 447 → 271 for BG and B6G, *m*/*z* 271 → 123 for baicalein, and *m*/*z* 271 → 153 for Genistein (IS), respectively. Other mass spectrum parameters included sheath gas (Arb) pressure, 20 psi, auxiliary gas (Arb) pressure, 2 psi; source voltage 3.5 KV, capillary voltage 2.5 KV, capillary temperature 275°C, capillary temperature 375°C.

### 2.5 Stability study in HBSS

The test was carried out by measuring the content of B, BG, and B6G before and after keeping in the HBSS solution at 37°C for 2 h. The time interval was selected according to the permeation experiment. The differences between the contents of the three samples before or after the incubation were calculated and expressed as the percentage of B, BG, and B6G remaining.

### 2.6 Evaluation of Caco‐2 cell monolayer

. The integrity of the cell monolayers was confirmed by measuring the Trans‐epithelial electrical resistance (TEER) before each permeation experiment.^16^ Millicell ERS‐2 voltmeter (Millipore) was used to measure the TEER value. TEER was also measured at various times during permeation experiments, including the start and finish times of the experiment.

The TEER values of Caco‐2 monolayers was measured and calculated as Equation (1):

(1) $$\text{TEER}\left(\Omega \cdot {\text{cm}}^{2}\right)=[\text{TEER}\left(\Omega \right)‐{\text{TEER}}_{\text{background}}\left(\Omega \right)]\times A\left({\text{cm}}^{2}\right)$$

Where TEER (Ω) is the electrical resistance across Caco‐2 monolayers directly read from the Millicell ERS‐2 epithelial voltmeter and TEER_background_(Ω) is only inserted across blank HBSS (no cells). *A* (cm^2^) is the area of the insert, 1.12 cm^2^.

To ensure the establishment of Caco‐2 cell monolayer model, it is necessary to verify multiple indicators. In this study, we also determined the activity of Alkaline phosphatase (As a brush‐edge marker enzyme differentiated during monolayer formation of Cac0‐2 cells). At 7, 14, 20 day during the culture, the culture medium of apical side and basolateral side of cell membrane was collected, and the activity of alkaline enzyme [Kim unit ·(100 ml)^−1^] was detected according to the Alkali phosphatase assay kit. According to the instructions of the kit, the Optical Density (OD) value was measured via a Varioskan multimode microplate spectrophotometer (Thermo Fisher Scientific) at 520 nm to determine the activity of alkaline phosphatase.

### 2.7 Permeation experiments

The permeation of samples was assessed in both directions at different concentrations. This experiment assessed the influence of different concentrations (%, v/v) of PEG400 on the permeation process of B, BG, and B6G. In addition, P‐GP inhibitor Verapami group (10 μM), MRP2 inhibitor MK‐571 group (10 μM), BCRP inhibitor KO143 group (10 μM) were set to assess the mediating effect of efflux transporter on BG and B6G transport.

Caco‐2 cell monolayers with qualified monolayer membrane integrity were used for drug permeability experiments. Aspirated and discarded the original culture medium in the transwell plate, added pre‐warmed (37°C) HBSS to wash 3 times, put it in the incubator for the last time and incubated for 30 min. The TEER value (≥500 Ω·cm^2^) of the monolayer was measured, proceed two‐way transport experiment. For the AP‐BL permeability (absorptive transport study), add 0.5 ml of samples (dissolved in HBSS) to the apical side (AP) as a supply chamber, and add 1.5 ml of pre‐warmed HBSS to the basolateral side (BL) as a receiving chamber. Alternatively, in the permeation direction of BL‐AP compartment (secretive transport study), add 1.5 ml of samples (dissolved in HBSS) to the BL side as a supply chamber, and add 0.5 ml of pre‐warmed HBSS to the AP side as a receiving chamber. After the above steps, put the transwell plate on the thermostatic oscillator (Changzhou Wanhe Instrument Manufacturing Co., Ltd.) at 60 r/min. And sample 100 µl from the receiving pool or 10 μl from the supply chamber at 30, 60, 90, and 120 min depending on the experiment, and make up the same volume of blank pre‐warmed HBSS. The sample taken from the transwell plate was precipitated with the 100 μl of methanol to precipitate the protein. After centrifugation at 16 000*g* for 15 min at 4°C, 70 μl of the supernatant was taken. And the content was determined by UPLC‐MS/MS method to calculate the apparent permeability coefficient (*P*_app_) and efflux ratio (ER).

The *P*_app_ and ER of Caco‐2 monolayers was measured and calculated as Equations ((2), (3), (4)):

(2) $$P_{\text{app}}=\frac{dQ/dt}{AC_{0}}$$

Where △*Q*/△*t* (ng/ml·s) is the rate of permeability (ng/ml·s for B, BG, B6G); *A* is for the membrane area (1.12 cm^2^); *C*_0_ (ng/ml/for B, BG, B6G) is the initial concentration of the sample in the supply chamber. The apparent permeability might need to be corrected because of the effect of the marked amount of baicalein glucuronide.^17^ Thus, the corrected permeability was calculated from the total rate of drug transport and the glucuronide excretion rate of bidirectional side divided by the membrane area (*A*) of the monolayer and the initial concentration of baicalein at the loading side (*C*_0_):

(3) $$P_{\text{corrected}}=\frac{\left(dQ/dt\right)+Aex+Bex}{AC_{0}}$$

(4) $$\text{ER}=P_{\text{appBL ‐ AP}}/P_{\text{appAP ‐ BL}}$$

Where *P*_appBL‐AP_ is the apparent permeability coefficient when the sample is added on the basolateral side; *P*_appAP‐BL_ is the apparent permeability coefficient when the sample is added on the apical side.

After the experiments, the TEER values of the monolayer were measured according to the detailed protocol described previously, to check the integrity of the Caco‐2 monolayer model.

### 2.8 Enzyme activity assay

In this experiment, the effect of PEG400 on the enzyme activities of UGT1A8 and UGT1A9 was evaluated by in vitro microsome incubation. The activity of UGT1A8 and UGT1A9 was determined by detecting the production of BG and B6G with B as substrate.

The incubation system contained 10 µl of 50 mM Tris‐HCl (pH 7.4) buffer, 10 µl of 5 mM MgCl_2_ solution, 10 µl of 0.2 mg/ml alamethicin (Aladdin Biochemical Technology), and 20 µl of intestinal microsomes (Xenotech). The incubation system was pre‐incubated on ice for 15 min prior to experiment. Then, added 20 µl baicalein (dissolved in 50 mM Tris‐HCl) and PEG400 (20 µl) or 50 mM Tris‐HCl (20 µl) to the incubation system. The reaction was initiated by adding 5 mM UDPGA (10 µl) and incubated at 37°C for 15 min. The whole reaction was operated in a 1.5 ml centrifuge tube. Finally, the reaction was terminated by adding cold acetonitrile (100 µl) containing the genistein (IS). All solutions were thoroughly mixed for 3 min, then the mixture was centrifuged at 4°C at 16,000*g* for 15 min to precipitate the proteins completely. The supernatant was transferred and 10 µl was injected into the UPLC‐MS/MS system.

Kinetics parameters were obtained by fitting the data with Michaelis‐Menten equation (Equation 5) using Prism 5.0 GraphPad Prism^®^ 9.

(5) $$v=\frac{V_{\max}\times \left[s\right]}{K_{m}+\left[s\right]}$$

where *v* (nmol/min/mg) is the velocity of metabolite formation, *V*_max_ (nmol/min/mg) is the maximum velocity, *K*_m_ (µM) is the Michaelis constant defined as the substrate concentration at half of *V*_max_, [S] (µM) is the substrate concentration

### 2.9 Statistical analysis

All experiments were performed at least in triplicate. Data are presented as means ± SD. The statistical analysis was performed using GraphPad Prism^®^ 9 (GraphPad Software). Statistical significance of differences in means was assessed using the Student's *t*‐test with *p* < .05 taken as significant and very significant at *p* < .01.

### 2.10 Nomenclature of targets and ligands

Key protein targets family's and ligands in this article are hyperlinked to corresponding entries in http://www.guidetopharmacology.org, the common portal for data from the IUPHAR/BPS Guide to PHARMACOLOGY (Harding et al., 2018), and are permanently archived in the Concise Guide to PHARMACOLOGY 2019/20 (Alexander et al., 2019).

## 3 RESULTS

### 3.1 Determination of samples

**FIGURE 1:**
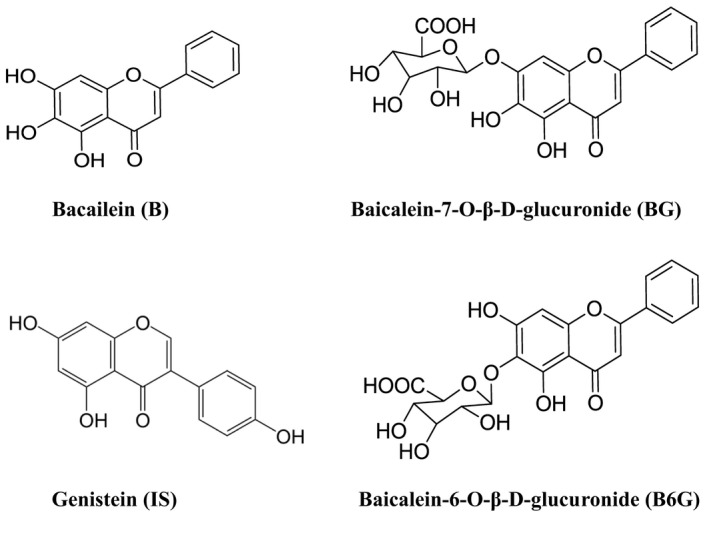
Chemical structures of baicalein, baicalein 7‐O‐β‐d‐glucopyranoside (BG), baicalein 6‐O‐β‐d‐glucopyranoside (B6G), and genisten (IS)

**FIGURE 2:**
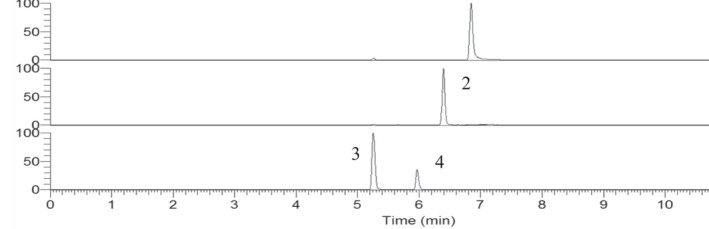
SRM chromatogram of each component. The sample was obtained from the permeability study and intestinal microsome regeneration system. Permeability samples obtained from two sides (apical side and basolateral side) at 2 h interval. Microsomal incubation samples after 15 min of incubation. (1) baicalein, (2) genisten, (3) baicalein‐7‐O‐β‐d‐glucopyranoside, (4) baicalein‐6‐O‐β‐d‐glucopyranoside

As shown in Figure 1, 2, the retention time for the four compounds were 5.27 min (BG), 5.95 min (B6G), 6.86 min (B) and 6.43 min (IS), it showed that the resolution of the analytical method is good. No matrix‐specific interfering peaks were observed.

### 3.2 Stability of samples

The stability of B, BG, and B6G in HBSS was studied and expressed as means ± SD of the three samples remaining with time after incubation at 37°C for 2 h. As shown in Table 1, the samples were stable during the incubation time (≥85%).

**TABLE 1:** The stability of B, BG and B6G in HBSS

Sample	Concentration/ng ml^−1^	Stability
B	150	98.11 ± 3.85
	300	99.70 ± 5.23
	600	98.43 ± 7.23
BG	150	93.35 ± 8.25
	300	99.20 ± 2.56
	600	100.61 ± 4.92
B6G	150	98.71 ± 6.52
	300	99.36 ± 6.23
	600	94.32 ± 5.33

Note

Results in mean ± SD from triplicate experiments.

### 3.3 Evaluation of Caco‐2 cell monolayer

In this study, two kinds of ways were used to confirm that the Caco‐2 cell monolayers were confluent and suitable for the permeation study: measured the TEER value of the Caco‐2 monolayer model; determined the activity of Alkaline phosphatase.

**FIGURE 3:**
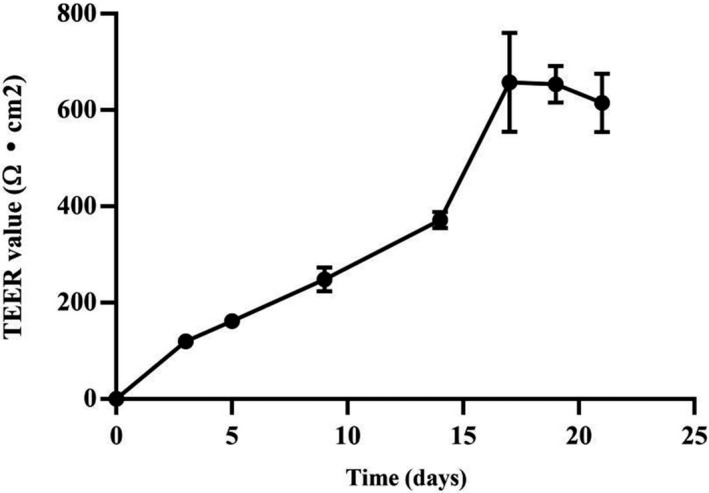
The characteristics of TEER value in different time. Data were expressed as means

**FIGURE 4:**
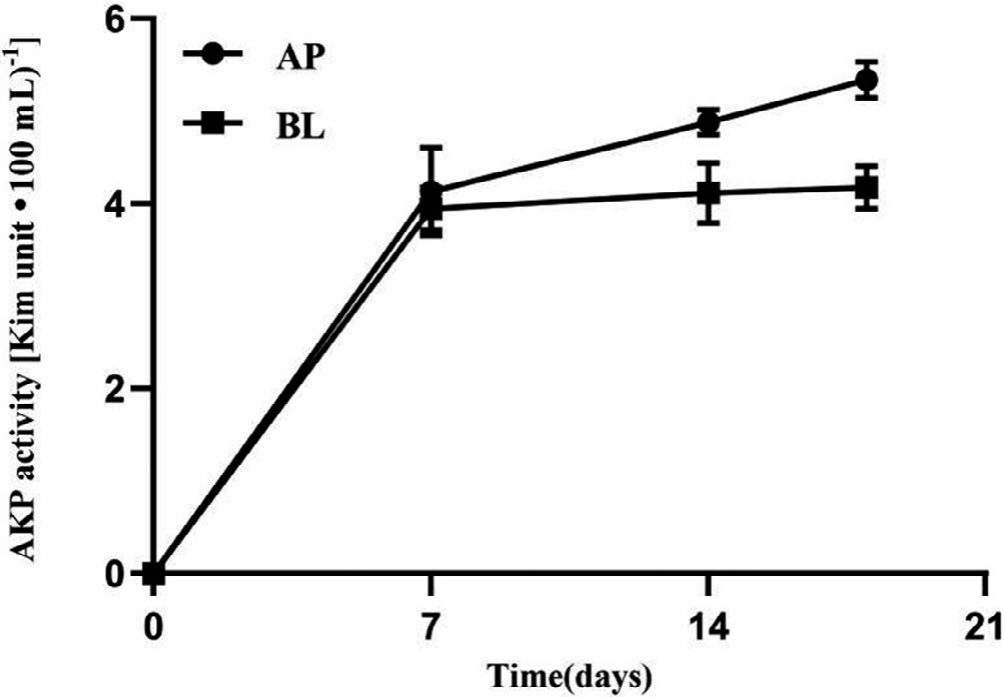
The differences of alkaline phosphatase activity in different time. The sample was obtained from apical side (AP) and basolateral side (BL). Data were expressed as means ± SD

Trans‐epithelial electrical resistance (TEER) Caco‐2 monolayer cultured in transwell plate was measured from day 3 to day 21. With the background resistance subtracted, as the culture time increased, the TEER value of the Caco‐2 cell monolayers was increased gradually, and reaching a peak at about 17 days, and gradually stabilized until 21 days (Figure 3). The TEER value during permeation experimentsremained stable thereby confirming integrity of the Caco‐2 cell monolayer junctions. On the hand, we have determined the activity of alkaline phosphatase (AKP) to assess the degree of cell differentiation. As shown in Figure 4, on day 20, the 3‐fold increase in AKP activity [Kim unit (100 ml) ^−1^] at apical side compared to basolateral side. It confirmed that Caco‐2 monolayer had differentiated and generated polarity.^18^ This complemented findings in the previously described junctional integrity evaluation that the Caco‐2 monolayer was suitable and ready for the permeability assay.

Finally, Caco‐2 monolayer model with a TEER value ≥500 (Ω·cm^2^) and a significant difference in phosphatase activity was selected for permeability permeation experiments.

### 3.4 The permeability study of baicalein in Caco‐2 monolayer

**FIGURE 5:**
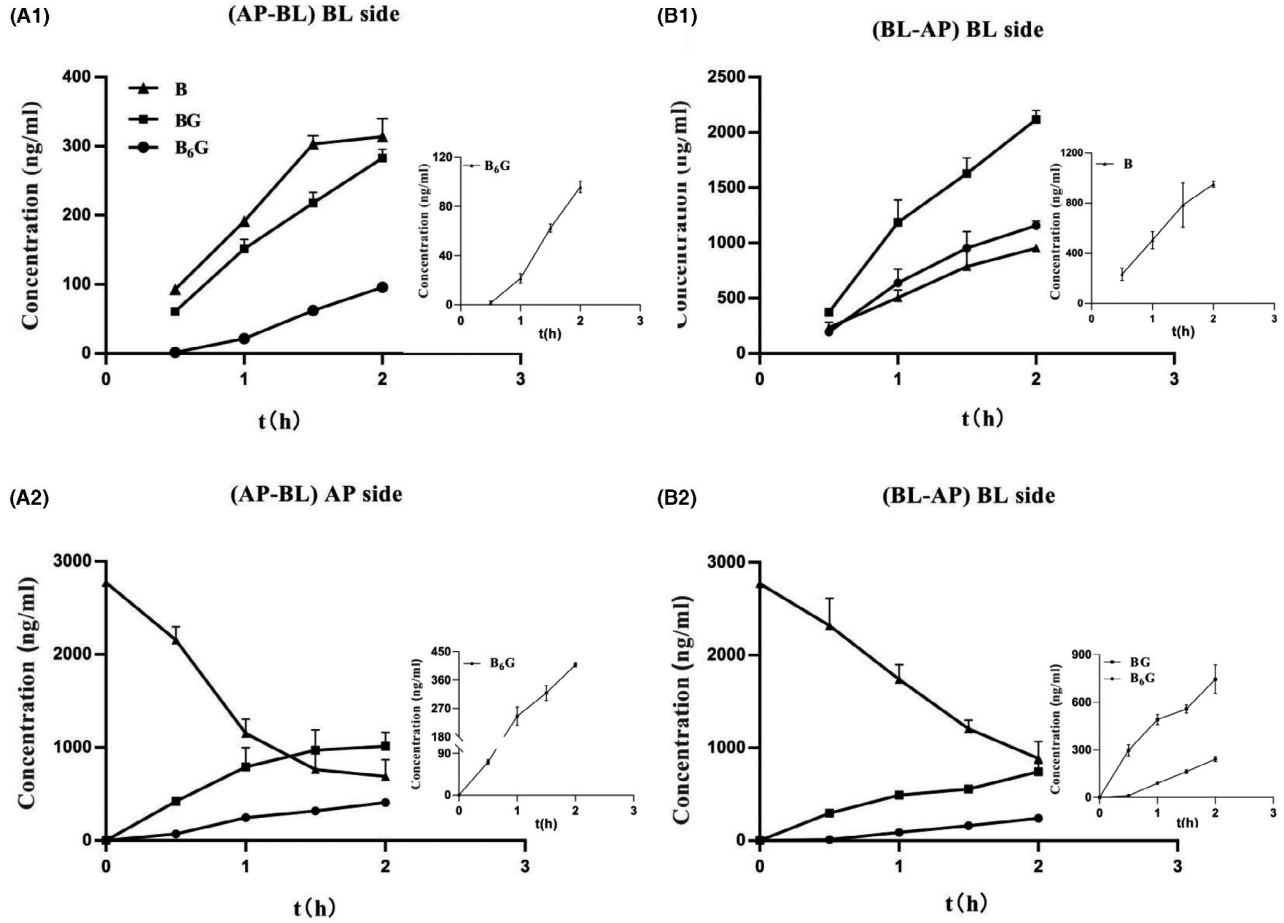
Baicalein at 10 μM was loaded to the apical side (AP‐BL) and basolateral side (B‐A) of the Caco‐2 cell monolayer. (A1) absorptive transport (AP‐BL) and (A2) secretive transport (BL‐AP) of B (triangle), BG (square), B6G (circle) across Caco‐2 cell monolayers. (B1) amount of B, BG, B6G in the (AP‐BL) apical side of absorptive transport (AP‐BL). (B2) amount of B, BG, B6G in the basolateral side of secretive transport (BL‐AP). The amounts of B, BG, B6G were determined at 0.5, 1, 1.5, and 2 h after incubation. Each data point represents the mean of three determinations, and the error bars represent the standard deviation

In this study, Caco‐2 monolayers were used for in vitro intestinal transport studies, and the intestinal permeability of baicalein was evaluated. As anticipated, in the penetration experiments of AP‐BL and BL‐AP, it was observed that the penetration of B, BG, and B6G gradually increased with time (Figure 5A1 and B1, respectively). This result indicated that B was metabolized by UGTs into glucuronide conjugates BG, B6G and transported across the monolayer. The penetration of BG and B6G increases linearly within 0–2 h. However, the linear penetration trend of B only occurred within 0–1.5 h, and the increasing trend of penetration weakened during 1.5–2 h (Figure 5A1 and B1). In the permeation experiment, we also collected a sample (10 µl) of the supply chamber, and observed the appearance of the glucuronic conjugates BG and B6G (Figure 5A2 and B1). By comparison, it was found that more BG and B6G were excreted into the AP side than into the BL side for both AP‐BL and BL‐AP transport experiments.

**FIGURE 6:**
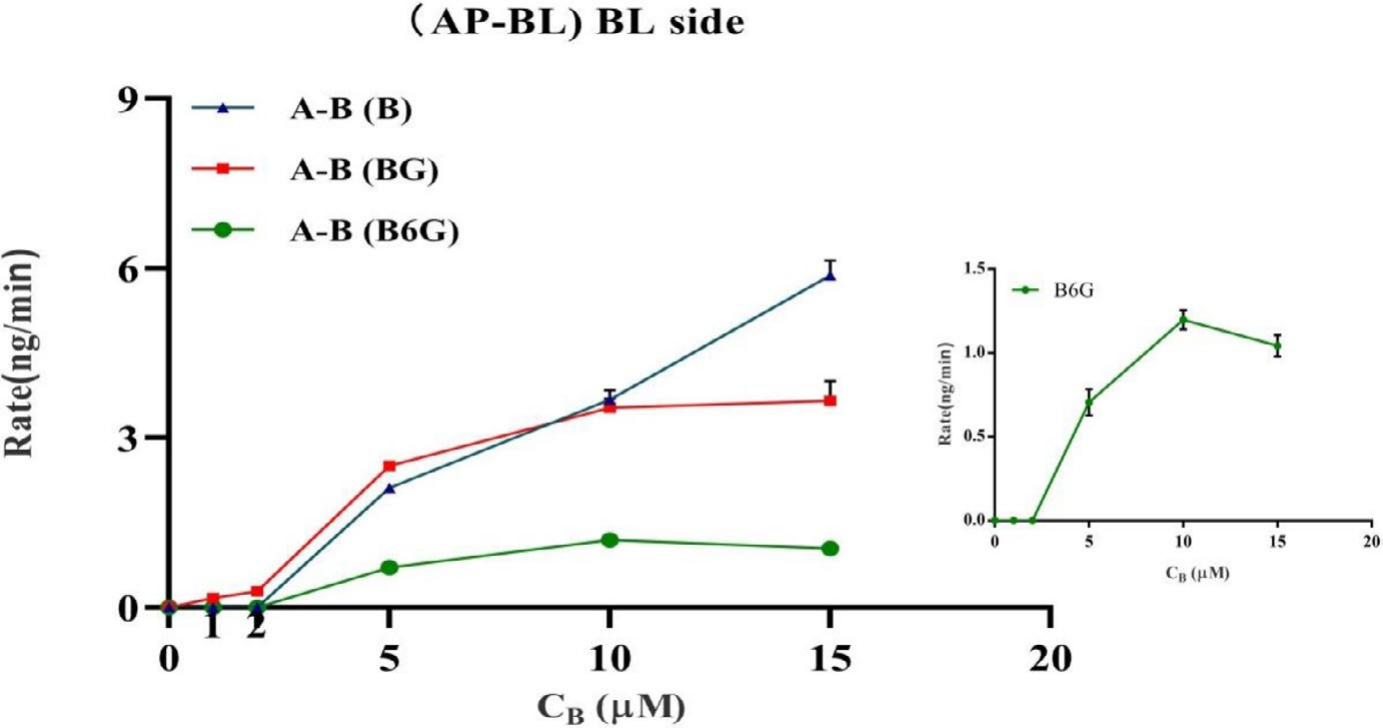
Concentration effects on the transport of B, BG, B6G in the Caco‐2 model. Baicalein at 1, 2, 5, 10 and 20 μM was loaded to the apical side (A–B) of Caco‐2 cell monolayer, the amount of B (triangle), BG (square), B6G (circle) from BL side was detected. Data were expressed by rate. The rate was represented the permeability amount of B, BG, and B6G per unit time (min)

**FIGURE 7:**
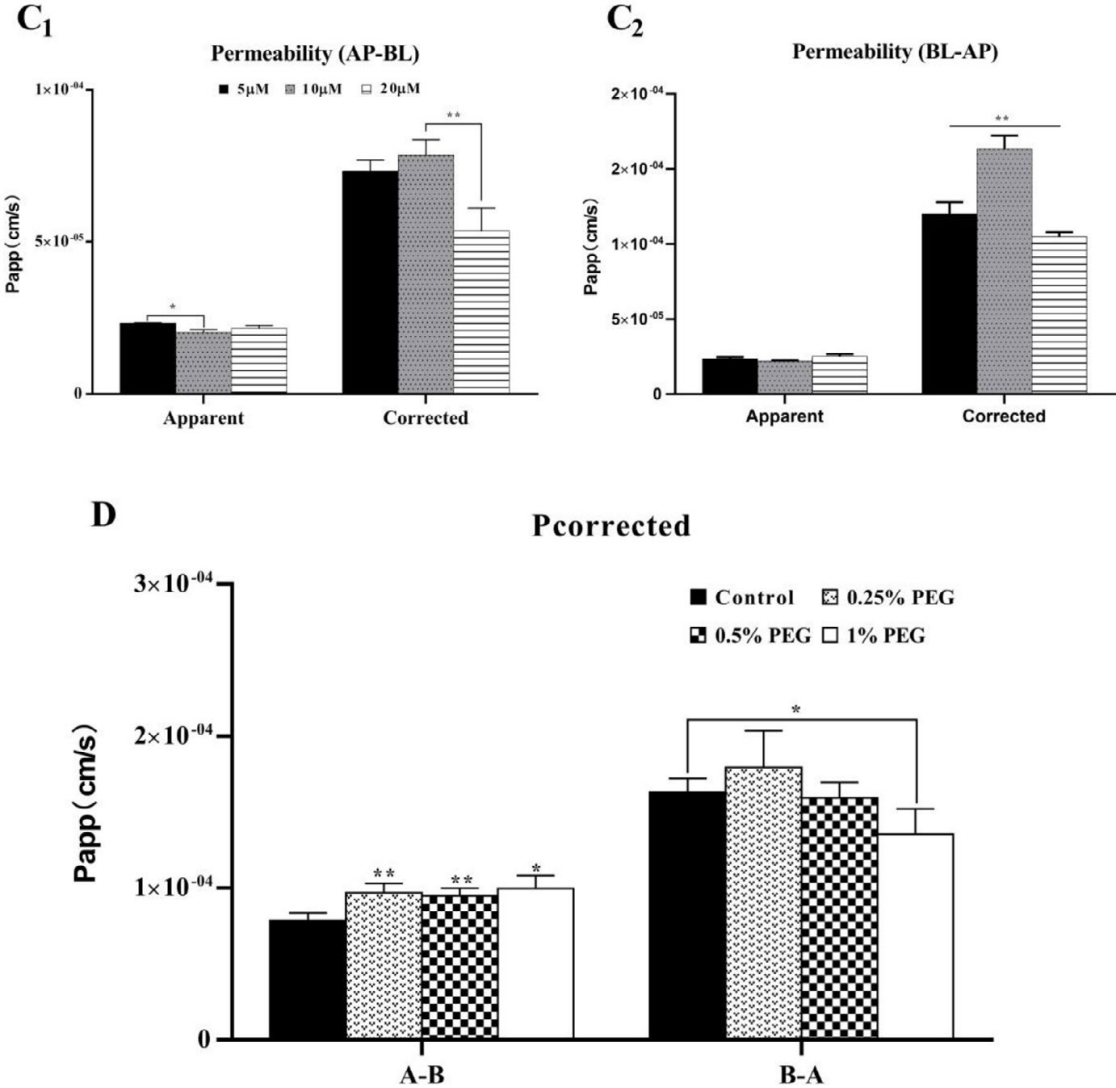
The apparent permeability and corrected permeability of the two‐way transport of baicalein. Baicalein at 5, 10, and 15 μM was loaded to the apical side (C_1_) and basolateral side (C_2_) of the Caco‐2 cell monolayer. Baicalein at 10 μM with PEG400 was loaded to the apical side (AP‐BL) of the Caco‐2 cell monolayer (D). The *P*_app_ value calculated as Equation $\left(P_{\text{app}}=\frac{dQ/dt}{AC_{0}},\;P_{\text{corrected}}=\frac{\left(\text{dQ}/dt\right)+Aex+Bex}{AC_{0}}\right)$. Data were expressed as the mean of three determinations, and the error bars represent the standard deviation. Significant differences (*p* < .05) between groups were marked by *. Very significant difference (*p* < .01) between groups are marked by **

In order to further determine the transmembrane absorption characteristics of baicalein, we evaluated the permeability characteristics of different concentrations of baicalein in Caco‐2 monolayers. The penetration of BG and B6G increased in a concentration‐dependent way when the concentration of B was between 0 and 10 μM on the AP side (absorption study). However, when the concentration of B is higher than 10 μM, the penetration of BG and B6G does not significantly change with the concentration, and there is saturation phenomenon (Figure 6). The *P*_app_ values of AP‐BL and BL‐AP permeability decreased gradually with the increase of the concentration when baicalein was loaded into the supply chamber at 5–10 μM. When the concentration of B is higher than 10 μM, it will increase with the increase of concentration (Figure 7C1 and C2). The corrected apparent permeability (Pcorrected) values for AP to BL (AP‐BL) were 73.18 × 10^−6^ ± 3.67 × 10^−6^ cm/s for 5 μM, 78.43 × 10^−6^ ± 5.17 × 10^−6^ cm/s for 10 μM, and 53.55 × 10^−6^ ± 7.60 × 10^−6^ cm/s for 15 μM. The corrected apparent permeability (*P*_corrected_) values for BL to AP (BL‐AP) were 120 × 10^−6^ ± 7.9 × 10^−6^ cm/s for 5 μM, 163.16 × 10^−6^ ± 9.04 × 10^−6^ cm/s for 10 μM, 104.77 × 10^−6^ ± 2.44 × 10^−6^ cm/s for 15 μM. *P*_corrected_ decreases due to the increase of baicalein concentration in the supply chamber after transport saturation (Figure 7C1 and C2).

**FIGURE 8:**
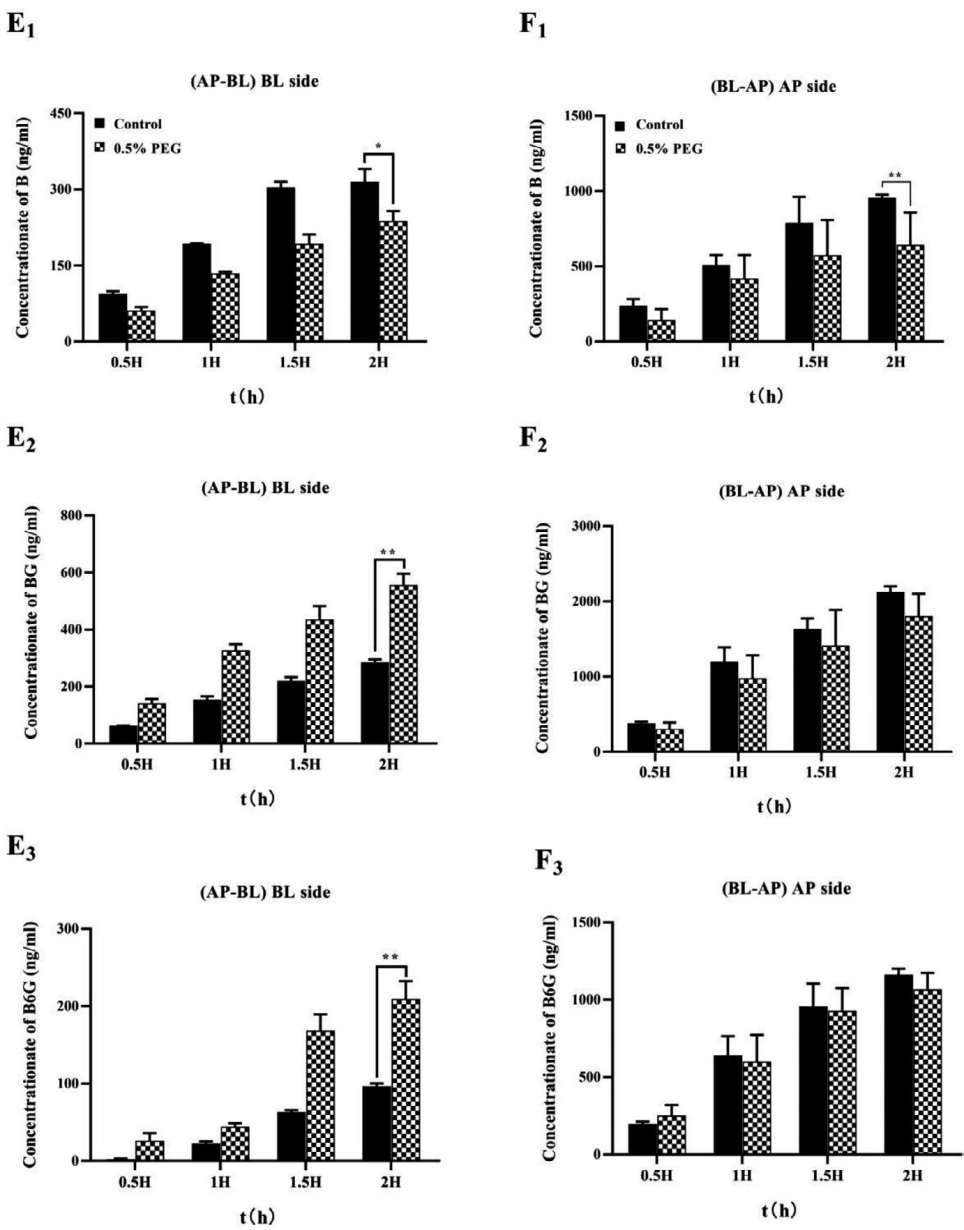
Effect of PEG400 on the two‐way transport of baicalein. Baicalein at 10 μM with 0.5% PEG400 was loaded to the apical side (AP‐BL) and basolateral side (BL‐AP) of the Caco‐2 cell monolayer. (E) Transport (AP‐BL) and (F) secretion (BL‐AP) of Baicalein across Caco‐2 cell monolayers. Control group was baicalein (10 μM) loaded in the supply chamber. 0.5% PEG group was baicalein (10 μM) with 0.5% PEG400 loaded in the supply chamber. Significant differences (*p* < .05) between groups were marked by *. Very significant difference (*p* < .01) between groups were marked by **

**FIGURE 9:**
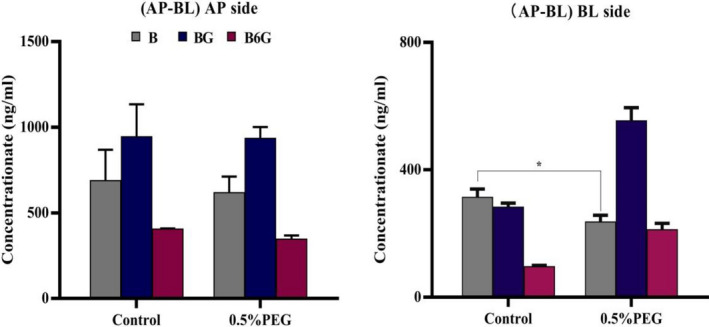
Effect of PEG400 on the absorptive transport of baicalein. Baicalein at 10 μM with 0.5% PEG400 was loaded to the apical side (AP‐BL) of the Caco‐2 cell monolayer. The samples of AP side and BL side were obtained after 2 h. Significant differences (*p* < .05) between groups were marked by *

We evaluated the effect of PEG400 on the permeability of baicalein in Caco‐2 monolayer by loading baicalein with PEG400 in the supply chamber. Compared with the control group, in the AL‐BL experiment, after 0.5% (v/v) PEG400 was added, the permeation of baicalein within 2 h was significantly decreased, while the permeation of BG and B6G was significantly increased (Figure 8E1–E3). In the BL‐AP experiment, after 0.5% (v/v) PEG was added, the penetration of baicalein within 2 h decreased significantly, but the penetration of BG and B6G did not show a significant difference (Figure 8F1–F3). It suggested that PEG400 could be involved in the metabolism of B in the Caco‐2 monolayer, and affect the penetration of glucuronide conjugates BG and B6G. By comparing the concentration of B on both sides of the monolayer after 2 h (AP‐BL), we found that there was no significant difference in the retention amount of baicalein (AP side) after the addition of PEG400 (Figure 9), which indicated that PEG400 did not change the intake of B.

**FIGURE 10:**
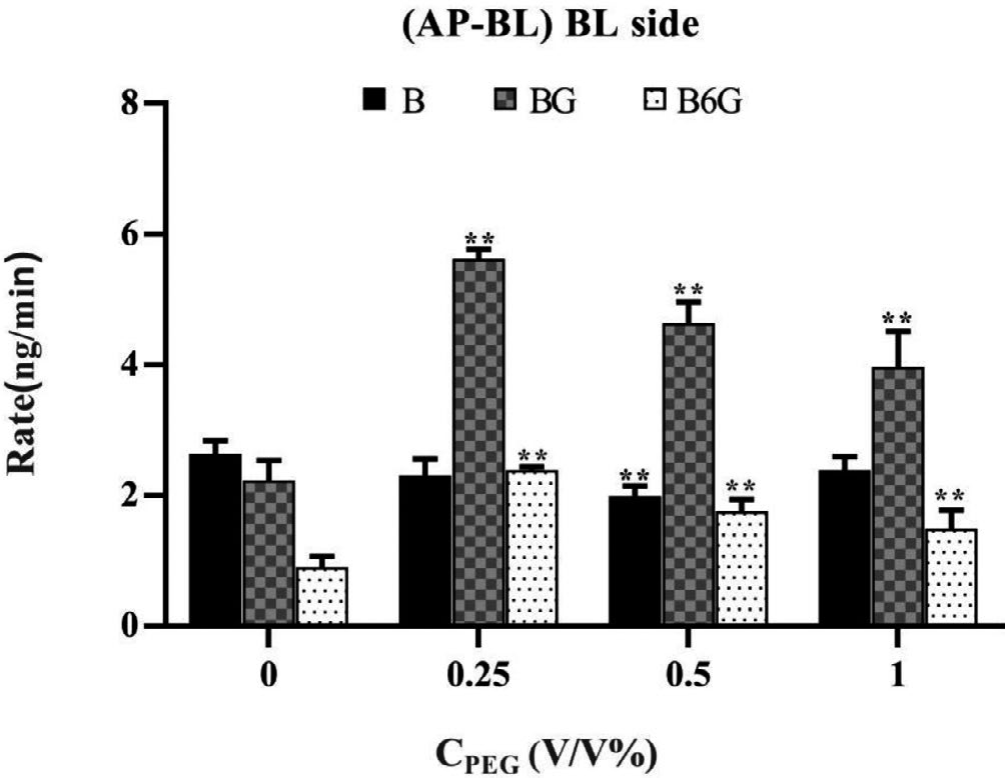
Effect of different PEG400 concentration on absorptive transport of baicalein. Baicalein at 10 μM with PEG400 was loaded to the apical side (AP‐BL) of the Caco‐2 cell monolayer. Data were obtained from BL sides and expressed by rate. The rate was represented the permeability amount of B, BG and B6G per unit time (min). Significant differences (*p* < .05) between groups were marked by *

In addition, we further evaluated the effect of PEG400 concentration on the permeability of baicalein in the Caco‐2 monolayer. As shown in Figure 10, different concentrations of PEG400 with B were added to the supply chamber. Compared with the control group, the permeability of BG and B6G both increased significantly, but not as a dose‐dependent increase. At a concentration of 0.5% PEG400, the permeability of baicalein decreased most significantly, but the permeability of BG and B6G was lesser than that of 0.25% PEG400 group. When 10 μM B was loaded on the AP side, the corrected apparent permeability (Pcorrected) was 100.27 × 10^−6^ ± 1.39 × 10^−6^ cm/s for 0.25% PEG400 group, 94.72 × 10^−6^ ± 4.51 × 10^−6^ cm/s for 0.5% PEG400 group, 99.69 × 10^−6^ ± 8.53 × 10^−6^ cm/s for 0.25% 1% PEG400 group. In the BL‐AP experiment, the Pcorrected was 186.49 × 10^−6^ ± 29.35 × 10^−6^ cm/s for 0.25% PEG400 group, 159.32 × 10^−6^ ± 10.1 × 10^−6^ cm/s for 0.5% PEG400 group, 135.35 × 10^−6^ ± 16.74 × 10^−6^ cm/s for 1% PEG400 group. Compared with control group, the corrected apparent permeability on the AP side will increase in the presence of PEG400, but it has not increased in a dose‐dependent manner. Compared with control group, the corrected apparent permeability on the AP–BL side increased in the presence of PEG400, but it had not increased in a dose‐dependent manner. The tendency of the corrected apparent permeability to increase in the presence of 0.25% PEG400 was the most significant. However, the trends on the BL–AP side were inconsistent. The corrected apparent permeability of the BL–AP side decreased significantly after adding a high concentration of 1% PEG400 (Figure 7D).

### 3.5 The permeability study of baicalein glucuronide conjugates in Caco‐2 monolayer

Based on the hydrophilicity of the glucuronic conjugate and the nature of the macromolecule, we speculated that the efflux transporters was involved in its excretion from the cell. In the two‐way permeation experiment of Caco‐2 monolayer, 5 μM BG and B6G were added to the supply chamber, and the *P*_app_ value was calculated. As shown in Table 2, *P*_appAL‐BL_ of BG was 6.52 × 10^−6^ ± 0.28 × 10^−6^ cm/s, and *P*_appBL–AL_ was 10.23 × 10^−6^ ± 0.37 × 10^−6^ cm/s. *P*_appAL–BL_ of B6G was 6.38 × 10^−6^ ± 0.26 × 10^−6^ cm/s, *P*_appBL–AL_ was 6.35 × 10^−6^ ± 0.55 × 10^−6^ cm/s. *P*_appAL–BL_ of BG and B6G are both lesser than *P*_appBL–AL_, and ER are 1.57 (BG) and 1.10 (B6G), respectively. It indicated that it may be actively transported during penetration and affected by efflux proteins. Therefore, in order to determine the main efflux transport protein that mediate the excretion of baicalein glucuronide BG and B6G, we used a variety of specific chemical inhibitors for different transport protein.

**TABLE 2:** The apparent permeability coefficient of BG and B6G on Caco‐2 monolayer

Experiment group	Sample	*P*_app_ (1 × 10^−6^ cm·s^−1^)		ER (*P*_BL‐AP_)/(*P*_AP‐BL_)
		Absorptive (AP‐BL)	Secretory (BL‐AP)	
BG	5 µM	6.52 ± 0.28	10.23 ± 0.37	1.57
	5 µM + 0.25%PEG400	9.91 ± 0.21**	10.24 ± 0.95	1.03
	5 µM + 0.5%PEG400	15.98 ± 0.11**	10.36 ± 0.05	0.65
	5 µM + 1%PEG400	14.37 ± 0.99**	8.24 ± 0.07**	0.57
	5 + 10 µM Ver	6.37 ± 0.53	10.52 ± 0.14	1.65
	5 + 10 µM MK571	13.24 ± 1.22**	11.38 ± 0.56*	0.86
	5 + 10 µM KO143	10.87 ± 0.4**	7.31 ± 0.90*	0.62
B6G	5 µM	6.38 ± 0.26	6.35 ± 0.55	1.01
	5 µM + 0.25%PEG400	6.74 ± 0.22	6.3 ± 0.98	0.93
	5 µM + 0.5%PEG400	6.15 ± 0.17*	4.39 ± 0.31*	0.71
	5 µM + 1%PEG400	7.53 ± 0.48*	4.24 ± 0.25**	0.56
	5 + 10 µM Ver	6.37 ± 0.15	6.26 ± 0.2	0.98
	5 + 10 µM MK571	6.91 ± 0.23*	4.56 ± 0.21**	0.65
	5 + 10 µM KO143	6.68 ± 0.06*	4.77 ± 0.09*	0.71

Note

Results in mean ± SD from triplicate experiments.

* *p* < .05, significant differences compared with control (5 μM) group.

** *p* < .05, very significant differences compared with control (5 μM) group.

In order to further determine the interaction between PEG400 and the efflux transporter, with different concentrations of PEG400 added, the two‐way permeability characteristics of baicalein glucuronide BG and B6G were evaluated, and *P*_app_ value was calculated. As shown in Table 2, the BG efflux rate (ER) under the intervention of 0.25% PEG, 0.5% PEG, and 1% PEG decreased from 1.57 to 1.03, 0.65, and 0.57, respectively, in a dose‐dependent manner. The B6G efflux rate (ER) under the intervention of 0.25% PEG400, 0.5% PEG400, and 1% PEG400 decreased from 1.01 to 0.93, 0.71, and 0.56, respectively, in a dose‐dependent manner.

### 3.6 The effect of PEG400 on UGT enzyme activity

**FIGURE 11:**
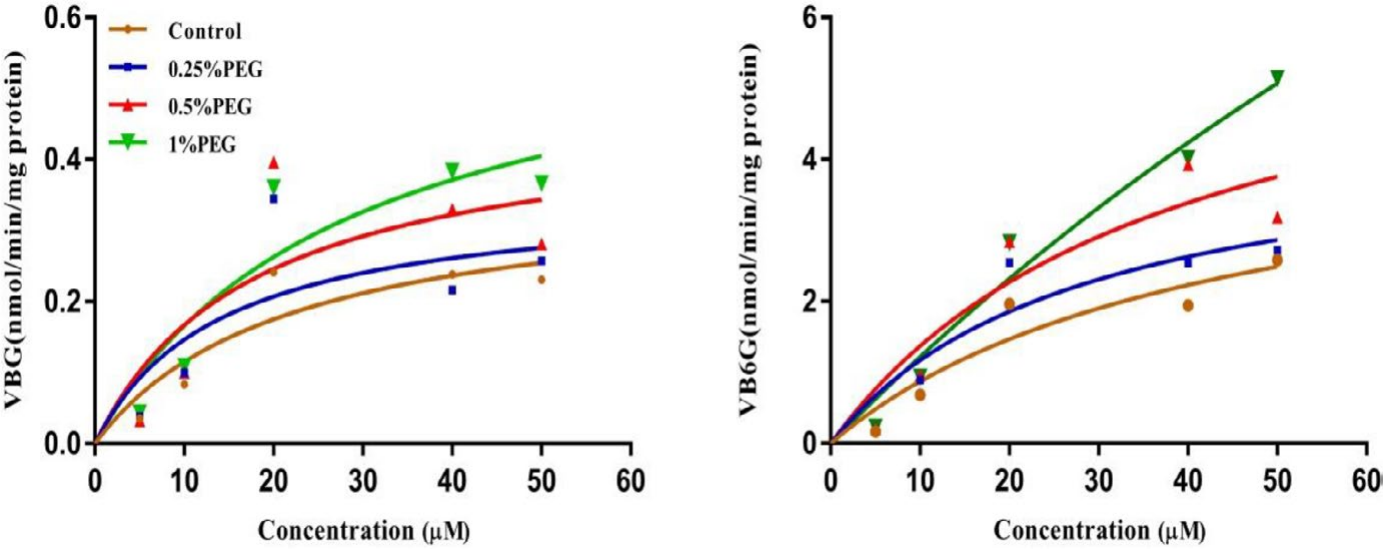
Effect of PEG400 on baicalein glucuronidation activity of UGT1A8 and UGT1A9. Baicalein was incubated in the microsomal system for 15 min as a substrate. UDP‐glucuronosyltransferase activity was expressed as glucuronide conjugate formed per minute per milligram protein (nmol/min/mg protein). All data were expressed as mean ± SD of triplicate experiments

In order to better understand the effect of PEG400 on the glucuronidation nature of UGT1A8 and UGT1A9, B was used as a substrate to determine the Michaelis constant (K_m_) and Maximum velocity (*V*_max_) of UGT1A8 and UGT1A9 within the concentration range of 0–50 µM (Figure 11). As shown in Table 3, the *V*_max_ and *K*_m_ of UGT1A8 were 4.64 ± 0.11 μmol/min/mg and 43.33 ± 2.63 µM, while the Vmax and Km of UGT1A9 were 0.37 ± 0.03 μmol/min/mg and 22.05 ± 3.74 µM, which indicated that the affinity of UGT1A9 with baicalein was higher than UGT1A8. In short, B was more likely to form BG in the microsomal incubation system. Under the intervention of low concentration of 0.25%PEG400, UGT1A8 *V*_max_ and *K*_m_ were 4.54 ± 0.40 μmol/min/mg and 28.44 ± 8.34 µM, UGT1A9 was 0.35 ± 0.01 μmol/min/mg and 14.34 ± 1.92 µM, which indicateed that PEG400 significantly decreased the affinity of the enzyme with the substrate. But under the intervention of high concentration of PEG400, it showed different characteristics. Under 0.5% PEG400 intervention, UGT1A8 *V*_max_ and *K*_m_ were 6.68 ± 0.29 μmol/min/mg and 38.86 ± 3.76 µM, UGT1A9 was 0.47 ± 0.05 μmol/min/mg and 18.26 ± 3.61 µM. Under 1% PEG400 intervention, UGT1A8 *V*_max_ and *K*_m_ were 24.78 ± 4.67 μmol/min/mg and 194.73 ± 48.82 μM, UGT1A9 was 0.63 ± 0.05 μmol/min/mg and 28.30 ± 3.33 μM. It showed that under the intervention of high concentration PEG400, it could be significantly increased the activity of the enzyme, but the affinity was significantly down‐regulated. Before and after PEG400 was added, the affinity of UGT1A8 to the substrate was always lower than that of UGT1A9.

**TABLE 3:** Kinetic parameters of baicalein glucuronidation by UGT1A8 and UGT1A9

Experiment group	Sample	*V*_max_ (nmol/min/mg protein)	*K*_m_ (µM)
BG (UGT1A9)	Control	0.37 ± 0.03	22.05 ± 3.74
	0.25% PEG400	0.35 ± 0.01	14.34 ± 1.92*
	0.5% PEG400	0.47 ± 0.05*	18.26 ± 3.61
	1% PEG400	0.63 ± 0.05**	28.30 ± 3.33*
B6G (UGT1A8)	Control	4.64 ± 0.11	43.33 ± 2.63
	0.25% PEG400	4.54 ± 0.40	28.44 ± 8.34*
	0.5% PEG400	6.68 ± 0.29**	38.86 ± 3.76
	1% PEG400	24.78 ± 4.67**	194.73 ± 48.82*

Note

Results in mean ± SD from triplicate experiments.

* *p* < .05, significant differences compared with control group.

** *p* < .05, very significant differences compared with control group.

## 4 DISCUSSION

As a traditional Chinese medicine of flavonoids, baicalein had been reported in modern pharmacological research to affect various biological processes such as cell proliferation, metastasis, apoptosis, and autophagy. It was a potential candidate for cancer treatment.^19^, ^20^ Baicalein can be well absorbed into the body from the intestine, and most of the absorbed baicalein is converted into glucuronide conjugates in the small intestine. This first pass effect is the main reason for the low bioavailability of xenobiotic drugs including baicalein after a single oral administration.^21^, ^22^

In this study, the intestinal absorption characteristics of baicalein were systematically evaluated in the Caco‐2 monolayer model. The UPLC‐MS/MS method was used to study the transmembrane penetration from the apical to the basolateral (AP‐BL) and basolateral to apical (BL‐AP). The permeation of baicalein on the Caco‐2 monolayer was strongly controlled by UGTs and efflux transport proteins. In the two‐way penetration experiment, baicalein would gradually permeate as time goes, and the permeability trend will weaken at 1.5–2 h, which could be caused by the concentration difference between the two sides of the cell monolayer gradually decreasing as the sample penetrated. It indicated that the way B enters the cell may be passive transport along the concentration gradient. We detected glucuronide conjugates BG and B6G in the receiving chamber and supply chamber at the same time, but they tended to flow out to the AP side (Figure 5). It showed that BG and B6G would penetrate into the supply chamber by efflux transport protein with reverse concentration trend, and the transport protein on the AP side played a dominant role. By comparing the permeability characteristics of different concentrations of B, we found that at a certain concentration, the permeability of BG and B6G did not increase with the increase of the concentration of B, which was a saturation phenomenon (Figure 6). It was due to limit by UGTs and the amount of expression in the cell itself. When saturation occurred, as the loading of baicalein in the supply chamber increased, the permeation of BG and B6G would not significantly change due to the saturation of production and efflux. When the low concentration of baicalein was loaded in the supply chamber, we only detected the penetration of BG, but B6G cannot be detected (Figure 6), suggesting that during the permeation process, B was more inclined to transform into BG.

The apparent permeability, as a classic parameter to evaluate the absorption characteristics of the drug on the Caco‐2 monolayer model, was calculated in this study. But baicalein *P*_app_ would be affected by the concentration of BG and B6G on both sides of the monolayer. We used the corrected apparent permeability (Pcorrected) to evaluate the absorption characteristics of baicalein in Caco‐2 monolayer. Since the penetration of baicalein was almost undetectable at low concentrations, it was not calculated. When 5–10 µM baicalein was loaded into the supply chamber, the metabolism was accelerated due to the increase in the concentration of baicalein in the supply chamber. And the Pcorrected was decreased. However, the corrected Pcorrected would increase due to the increase in the overall BG and B6G excretion rate (Figure 7C1 and C2). After BG and B6G are saturated, the uncorrected apparent permeability does not change significantly with the addition of baicalein in the supply chamber. The Pcorrected was decreased because the overall BG and B6G have no variation (Figure 7C1 and C2). The corrected apparent permeability was used to reflect the bioavailability of the drug. The above results indicated that the metabolism of UGTs was the main reason for the low oral bioavailability of baicalein, and it decreased significantly as the oral dose increased.

The Caco‐2 monolayer had been widely used in the identification of substrates, inhibitors and inducers of intestinal transport proteins^23^ The verapamil (P‐gp transport protein inhibitor), MK571 (MRP transport protein inhibitor) and KO143 (BCRP transport protein inhibitor) was added to sifting out for efflux transporters that mediate BG and B6G. The addition of verapamil did not change the apparent permeability of the two‐way penetration of BG and B6G. However, the addition of MK571 and KO143 decreased the efflux ratio of BG and B6G (Table 2). It showed that MRP and BCRP mediated in the efflux of BG and B6G, but P‐gp did not. Transport proteins were located on different sides of the cell membrane. As AP side transport proteins(MRP2 and BCRP)were the main mediator, BG and B6G were more likely to be excreted to the AP side.

**FIGURE 12:**
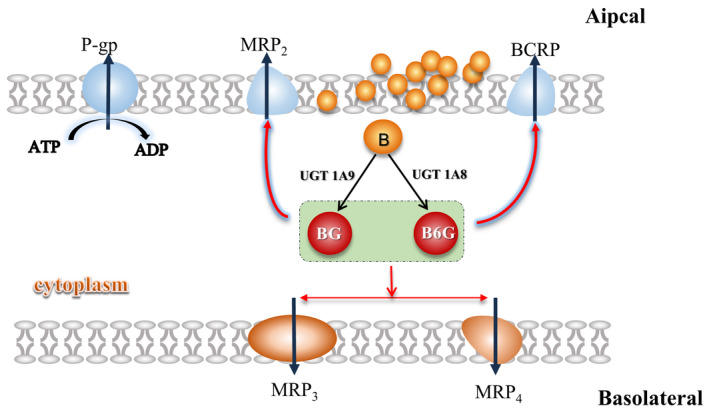
The schematic representation of the intestinal disposition pathways of baicalein

We had clarified the characteristics of baicalein absorption in the intestine. After B enters the cell, it was metabolized into glucuronide conjugates BG and B6G under the action of UGTs on the endoplasmic reticulum membrane, and then mainly excreted to different sides by acting of MRP and BCRP (Figure 12).

Interactions occur when a substance alters the reaction of drugs that are co‐administered, the most common being drug‐drug interactions (DDI). Drug metabolism enzymes and transport proteins have been widely reported as the main mediators of DDI.^24^, ^25^ There had been a large amount of evidence that many excipients were not pharmacologically inert, and the absorption, distribution, metabolism and elimination (ADME) of active pharmaceutical ingredients might be altered by the regulation of excipients on their metabolism and transport.^26^ In this study, we confirmed the interaction of excipient PEG400 with the metabolic enzymes and transport protein in the Caco‐2 monolayer model. Since saturation had already occurred at 10 μM, this could have reached the limit of the effect of metabolic enzymes and transport proteins in the Caco‐2 monolayer model. Therefore, the effect of PEG400 on drug absorption under limiting conditions was explored to clarify the interaction of PEG400 with metabolic enzymes and transport proteins.

Under the intervention of 0.5% PEG400, in the AL‐BL side penetration experiment, the penetration of baicalein was significantly decreased within 2 h, but the penetration of BG and B6G were significantly increased (Figure 8E1–E3). We found that the addition of PEG400 did not change the concentration of B, BG, and B6G in the supply chamber by comparing the changes of drug concentration on both side (Figure 9). In the BL‐AL side penetration experiment, the intervention of PEG400 not only decreased the penetration of baicalein, but also did not change the efflux of BG and B6G to AP (Figure 8F1–F3). It indicated that the addition of PEG400 did not change the uptake of drugs by cells, but only affected the metabolic process of UGTs on B, and accelerated the penetration of BG and B6G from AP to BL. Therefore, we further verified the effect of PEG400 on the efflux of BG and B6G. The addition of PEG400 significantly decreased the efflux ratio of BG and B6G to AP, and gradually decreased as the dose of PEG400 increased (Table 2). It indicated that PEG400 has an inhibitory effect on the efflux transport protein after excluding the influence of metabolic enzymes. Previously, BG and B6G have been confirmed as substrates of the efflux transporter MRP and BCRP in my study, and the AP side transporter plays a dominant role. It suggested that PEG400 affected the efflux of BG and B6G by interacting with MRP and BCRP. However, in the normal absorption process, metabolism and transport are involved. Perhaps at 0.5% concentration, UGTs metabolism of drugs was more likely to be affected, so there was no significant change in drug efflux to AP side. The excess BG and B6G penetrated to BL side through MRP3 and MRP4.

We also evaluated the effect of PEG400 concentration on the permeability of baicalein to clarify the effect of different concentrations of PEG400 on metabolic enzymes and transport proteins. The three concentrations of low, medium, and high showed inconsistent impact results (Figure 10). In the absorption experiment (AL‐BL), we found that under the intervention of low concentration of PEG400 (0.25%), the penetration of BG and B6G generated was the largest. However, the penetration of baicalein was higher than that of the medium concentration PEG400 group (0.5%). Under the intervention of the high concentration PEG400 group (1%), the penetration of B was the maximum, and the penetration of BG and B6G was the lowest. By comparing the *P*_corrected_, we found that different concentrations of PEG400 group significantly increased the *P*_corrected_ value of AP‐BL penetration experiments. This is because the additionEG400 increased the overall drug concentration, confirming that PEG400 can indeed increase the bioavailability of baicalein in the body. But on the other side, in the BL‐AP experiment, there was no significant difference (Figure 7D). This may be due to the passive diffusion process of BG and B6G, and the excessive drug concentration on the BL side is not conducive to the migration of BG and B6G to the BL side. This also be one of the reasons why there is no significant difference in the permeability of BG and B6G on the BL side of the 0.5% PEG400 group (Figure 8F1–F3). Excessive BG and B6G permeated into the AP side along the concentration gradient and offset the inhibitory effect of 0.5%PEG400 on efflux protein. Under the intervention of the high concentration PEG400 group (1%), the corrected *P*_corrected_ decreased significantly. This should be due to the reason that the high‐concentration PEG400 strongly inhibits the efflux protein and excessively generated BG and B6G accumulate in the cell, resulting in the overall level of BG and B6G on both sides of monolayer decreased.

BG and B6G are the main glucuronide metabolites of baicalein, which are mainly regulated by UGT1A9 and UGT1A8.^27^, ^28^ The acceleration of glucuronide metabolism depends on the increase of enzyme activity. Therefore, we further established an in vitro incubation system to confirm the effect of PEG400 on the metabolic activity of UGT1A8 and UGT1A9 in the intestinal microsome.

By matching the data from the incubation system to the Michaelis‐Menten equation, *V*_max_ and *K*_m_ are calculated. *V*_max_ reflected the activity of the enzyme. *K*_m_ reflected the affinity between enzyme and substrate and determines the direction of drug metabolism. The addition of medium and high concentrations of PEG400 significantly increased the *V*_max_ value with a dose‐dependent manner. But when PEG400 (0.25%) was added, *V*_max_ did not change significantly, but the *K*_m_ value of the enzymatic reaction was decreased (Table 3). These results showed that PEG400 could increase enzyme activity and accelerate the metabolism of metabolic enzymes. And a certain concentration of PEG400 could change the affinity of the enzyme and the substrate which affect the tendency of the substrate to transform. It also explained that when 0.25% PEG400 was loaded on the AP side, the generation of BG and B6G was significantly higher than that of the high‐concentration PEG400 group due to the increased affinity of UGT1A8 and UGT1A9. Before and after the addition of PEG400, the *K*_m_ value of the metabolic enzyme UGT1A9 was always lower than UGT1A8 (Table 3). It indicated that B was more likely to react with UGT1A9, led to the original evaluation of B penetration characteristics, only the penetration of BG can be detected at low concentrations (Figure 6).

In previous studies, PEG400 was reported as an inhibitor of liver uptake transporters.^29^ This suggests that the effect of PEG400 on baicalein may be due to interaction with the transporter. However, the metabolic process of B glucuronidation is carried out on the intracellular mitochondria. Although the interaction of PEG400 with the efflux transporter P‐gp has been described,^30^ but it had been found in our research that BG and B6G were effluxed as substrates of MRP and BCRP. Therefore, in our research, our focus is mainly on the interaction of PEG400 with the efflux transporter MRP and BCRP. In the in vitro enzymatic kinetic study, B was used as a substrate to confirm the interaction of PEG400 with UGT1A8 and UGT1A9. The innovative point of our research is to reveal the interaction between PEG400 and UGT1A family members (UGT1A8 and UGT1A9), which is different from previous studies.^31^ Unfortunately, we have not been able to explain the mechanism by which PEG400 interacts with transport enzymes and metabolic enzymes. In the system of solution containing PEG400, the process of metabolism and transportation of B is changed. The flexible chain of PEG can produce steric hindrance, which may hinder the binding of drug and protein. This is a point that we must focus on in the next stage of research. This is the focus of a deeper explanation of the interaction between PEG400 and transporter.

In conclusion, the pharmaceutical excipient polyethylene glycol 400 has a certain interaction with UGTs and efflux transport proteins. It will lead to changes in the absorption characteristics of baicalein. In preparations containing PEG400, we must consider the effects of several UGTs and efflux proteins on the in vivo kinetics of the main drug, including UGT1A8, UGT1A9, MRP2, MRP3, MRP4 and BCRP. The most important thing is that the different dosage of PEG400 in the drug preparation will have different effects, which is worthy of our further consideration.

## DISCLOSURE

The authors declare that there are no conflict of interests.

## AUTHOR CONTRIBUTIONS

Participation in research design: Siyuan Cao, Xiuli Gao, Rongping Zhang. Conducted experiments: Siyuan Cao, Min Zhang, Mei Zhao and Minyan Yuan. Contributed new reagents or analytic tools: Shuo Zhang. Performed data analysis: Siyuan Cao, Dan yang and Penjiao Wang. Wrote or contributed to the writing of the manuscript: Siyuan Cao and Xiuli Gao.

## ETHICS STATEMENT

This manuscript is not published elsewhere.

## Data Availability

The data that support the findings of this study are openly available in figshare.
